# Dynamic transcriptome unveils transcriptional network associated with chlorophyll degradation in tobacco leaves under high humidity and temperature

**DOI:** 10.3389/fpls.2026.1758730

**Published:** 2026-02-11

**Authors:** Yike Su, Xiaodong Hu, Xiaojuan Yang, Jiaen Su, Yanming Yang, Yi Chen, Yonglei Jiang, Nan Shi, Mengxia Li, Bo Zhang, Binbin Hu

**Affiliations:** 1 Yunnan Academy of Tobacco Agricultural Sciences, Kunming, China; 2 College of Agriculture and Biotechnology, Zhejiang University, Hangzhou, China; 3 Tobacco Monopoly Bureau of Yunnan Province, Kunming, China; 4 Tobacco Monopoly Bureau of Dali State, Dali, China

**Keywords:** chlorophyll degradation, high humidity, nitrogen imbalance, postharvest curing, transcriptional network, transcriptome

## Abstract

**Introduction:**

Chlorophyll degradation is a key determinant of postharvest quality in flue-cured tobacco. Excessive nitrogen application increases chlorophyll accumulation, leading to green discoloration or mottling and reducing market value after curing.

**Methods:**

This study investigated the effects of environmental humidity on chlorophyll degradation through gene ontology (GO) enrichment analysis, differentially expressed genes (DEGs) analysis, and correlation analysis.

**Results:**

At 93% relative humidity (RH), the rate of chlorophyll degradation was approximately twofold higher than that observed at 79% RH, particularly in nitrogen-imbalanced tobacco leaves. Transcriptome analysis of 63 samples revealed stage-specific expression patterns of chlorophyll catabolic genes (CCGs) under different curing conditions. PAO2 showed pronounced expression at 12 h, whereas CLH6 exhibited high expression at 18 h under high humidity. Under high-temperature stress during curing, SGR6 and CLH5 were strongly induced. Correlation-based transcriptional network analysis indicated that multiple transcription factor families, including ERF, NAC, MYB, and WRKY, were closely associated with CCG expression in response to humidity and temperature changes. Together, these findings suggest a complex transcriptional landscape linking environmental conditions with chlorophyll degradation during tobacco curing.

**Discussion:**

The identified genes and transcriptional associations provide potential molecular candidates for future functional validation and the improvement of curing compatibility in tobacco.

## Introduction

Chlorophyll (Chl) is the central photosynthetic pigment in plants, playing an essential role in light absorption and energy transfer to reaction centers (Li et al., 2024). In higher plants, chlorophyll exists mainly as chlorophyll a and chlorophyll b, and its degradation is a tightly coordinated biological process during leaf senescence, fruit ripening, seed development, and responses to environmental stresses (Tanaka and Ito, 2025). Chlorophyll catabolism involves multiple enzymatic steps, including The interconversion of Chl b to Chl a and the subsequent breakdown to Chl a into non-colored catabolites through the pheophorbide an oxygenase (PAO) and red chlorophyll catabolite reductase (RCCR) pathway (Hoertensteiner, 2006; Hoertensteiner and Kraeutler, 2011; Nakajima et al., 2012; Sato et al., 2015; Hauenstein et al., 2016; Kraeutler, 2016; Hu et al., 2021).

Previous studies have demonstrated that chlorophyll degradation is associated with complex transcriptional programs involving multiple transcription factor families, such as NAC, WRKY, ERF, bHLH, bZIP, and MYB (Shan et al., 2021; Song et al., 2023; D’Incà et al., 2023; Li et al., 2025; Ma et al., 2025). Functional studies in model and horticultural plants have shown that specific transcription factors can influence the expression of chlorophyll catabolic genes (CCGs) under developmental cues or environmental stresses, including temperature and light conditions (Zhu et al., 2015; Cheng et al., 2020; Wei et al., 2023; Pei et al., 2025). For example, ANAC019/055/072 activate CCGs such as NYE1/SGR1, NYE2/SGR2, and NYC1 (Zhu et al., 2015; Major et al., 2017). MdERF17 interacts with and is phosphorylated by MdMPK4-14G, blocking chlorophyll degradation (Wang et al., 2022). Together, these findings highlight that chlorophyll degradation is not a linear process but is embedded within a broader transcription landscape to environmental signals.

Tobacco (*Nicotiana tabacum*) is an economically important crop cultivated worldwide, and chlorophyll degradation during flue-curing is a critical determinant of cured leaf color and overall quality (Kuai et al., 2018). During curing, chlorophyll is progressively degraded as leaves transition from green to yellow brown stage (Song et al., 2010). Nevertheless, tobacco production frequently encounters quality defects such as undercuring and motting, which manifest as greenish discoloration and substantially reduce market value. These defects are often associated with excessive nitrogen fertilization, which disrupts chlorophyll metabolism before and after curing.

Two nitrogen-related physiological disorders are particularly problematic: Laohan (nitrogen-overfertilized tobacco) and Fanqing (greening reversion tobacco). Laohan results from excessive nitrogen application during the early growth, leading to over-vegetative leaves with delayed maturity, resulting in slow yellowing during flue-curing. Fanqing typically occurs after rainfall following topping, when the residual soil nitrogen is reabsorbed by the plant, triggering chlorophyll resynthesis and causing the mature leaves to regain green pigmentation. Suboptimal curing conditions, especially inappropriate temperature and humidity, further exacerbate these issues by interfering with chlorophyll degradation during postharvest processing.

Although previous studies have shown that curing temperature and humidity strongly influence chlorophyll loss in tobacco leaves (Li et al., 2021; Meng et al., 2024), how nitrogen-affected leaves with distinct physiological backgrounds respond transcriptionally to these environmental conditions remains poorly characterized. In particular, comparative analyses of Laohan and Fanqing leaves under controlled curing environments are lacking, and the transcriptional features associated with temperature- and humidity-driven chlorophyll degradation during curing are still unclear.

In this study, we investigated how postharvest temperature and humidity conditions are associated with chlorophyll degradation dynamics in nitrogen-affected tobacco leaves during the curing process. Using Laohan and Fanqing leaves as materials, we combined physiological measurements with transcriptome analysis to characterize chlorophyll degradation patterns and identify transcriptional responses linked to environmental variation. This work provides a transcriptome-level framework for understanding chlorophyll degradation during tobacco curing and offers insights relevant to the optimization of postharvest curing strategies.

## Results

### Increased relative humidity is associated with accelerated chlorophyll degradation during postharvest tobacco flue-curing

In order to investigate the impacts of humidity on chlorophyll degradation during flue-curing, three types of tobacco with distinct chlorophyll contents resulting from various nitrogen fertilization regimes were selected (**Table 1**). These included control tobacco, Laohan tobacco, and Fanqing tobacco. Leaves from each type were subjected to two different humidity (RH) conditions during curing (Treatment 1: 79% RH; Treatment 2: 93% RH), while temperature was maintained constant (**Supplementary Figure S1**).

**Table 1:** Nitrogen fertilization schedule for tobacco cultivation (kg/ha).

Application timing	Control	Laohan	Fanqing
Transplantation (Base fertilizer)	18	18	18
1 week after transplantation	18	18	18
2 weeks after transplantation	18	18	18
3 weeks after transplantation	18	27	18
4 weeks after transplantation	10.5	45	–
5 weeks after transplantation	–	16.5	–
At topping	–	–	27
1 week after topping	–	–	27
2 weeks after topping	–	–	16.5
Total Nitrogen Applied	82.5	142.5	142.5

**Figure 1:**
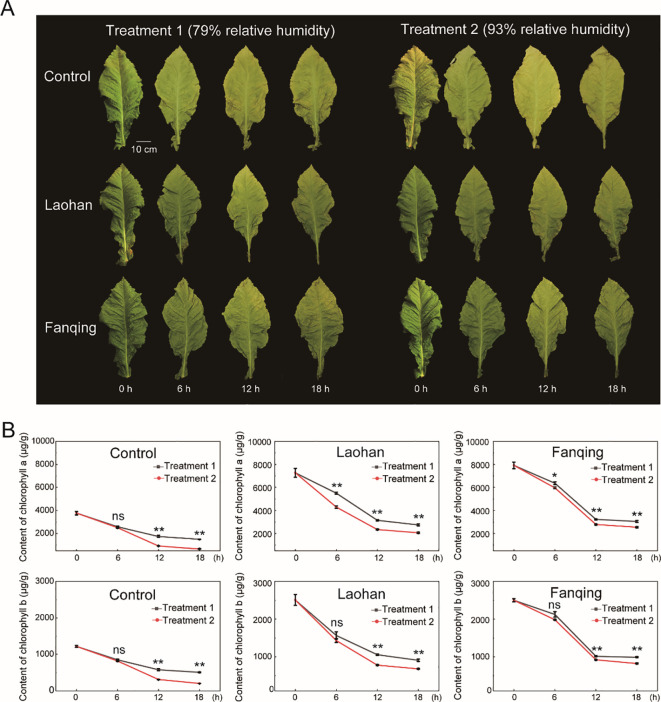
Changes in chlorophyll content during the tobacco flue-curing under humidity conditions. **(A)** Representative photographs of tobacco leaves during the curing process under two humidity treatments: Treatment 1 (79% RH) and Treatment 2 (93% RH). **(B)** Changes in chlorophyll a and chlorophyll b contents in three tobacco types during the curing process under different humidity conditions. Data represent the mean ± SD of three independent biological replicates (n = 3). Statistical significance between humidity treatments at each time point was determined using a two-tailed Student’s *t*-test. *P < 0.05, **P < 0.01.

As shown in **Figure 1A**, all tobacco leaves exhibited a typical green coloration at the onset of curing (0 h), followed by progressive yellowing as curing processed. Compared with the control leaves, both Laohan and Fanqing tobacco leaves displayed a visible darker green color at 0 h, consistent with their higher initial chlorophyll contents. Quantitative analysis confirmed that both chlorophyll a and chlorophyll b contents were significantly higher in Laohan and Fanqing leaves than in the control group at the initial time point (**Figure 1B**). Specially, the chlorophyll a content in control leaves was approximately 4000 μg/g at 0 h, whereas values of 7380 μg/g and 8140 μg/g were observed in Laohan and Fanqing leaves, respectively (**Figure 1B**). Moreover, the content of chlorophyll b is approximately 45% and 49% of that measured in Laohan and Fanqing leaves, respectively.

Chlorophyll content declined continuously in all three tobacco types throughout the curing process, regardless of humidity treatment. However, the rate of chlorophyll loss differed markedly between the two humidity conditions. Under high humidity (Treatment 2, 93% RH), chlorophyll degradation occurred more rapidly than under moderate humidity (Treatment 1, 79% RH) (**Figure 1B**). In the control group, chlorophyll a and chlorophyll b contents decreased to approximately 40% of their initial levels after 18 h under Treatment 1, whereas they declined to about 17% under Treatment 2. Similarly, in Laohan tobacco, chlorophyll a content decreased from 7380 μg/g at 0 h to 3108 μg/g under Treatment 1 and to 2184 μg/g under Treatment 2 at 18 h. In Fanqing tobacco leaves, chlorophyll a content reached 3354 μg/g under Treatment 1 and 2672 μg/g under Treatment 2 at the same time point. These findings indicate that elevated relative humidity is closely associated with a faster decline in chlorophyll content during tobacco flue-curing, particularly in leaves with high chlorophyll levels.

### Transcriptome-wide response of tobacco leaves to different humidity conditions during flue-curing

To characterize transcriptome-wide responses of tobacco leaves to different humidity conditions during flue-curing, RNA sequencing was performed on samples collected throughout the curing process. A total of 63 RNA-seq libraries were generated, yielding approximately 62 million clean reads and 586.6 Gb of high-quality sequencing data (**Supplementary Table S1**). Samples at 0 h three tobacco types prior to curing and were therefore excluded from humidity-related. Samples collected at 0 h represented three tobacco types prior to curing and were therefore excluded from humidity-related transcriptomic comparisons. The remaining 54 samples were subjected to downstream analyses and grouped according to humidity treatment, with temperature maintained constant at 38 °C during curing.

**Figure 2:**
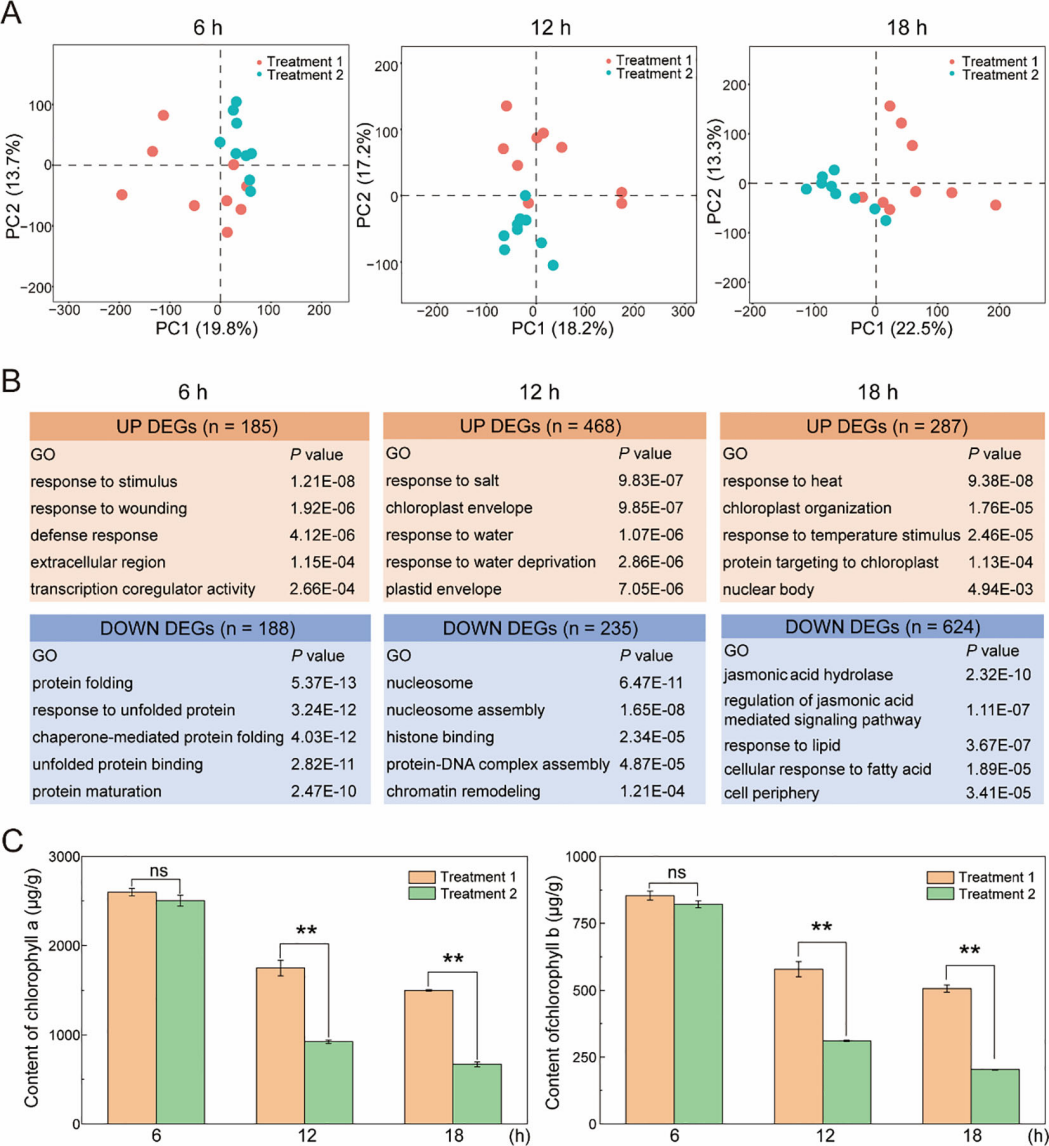
Transcriptomic and physiological response of tobacco leaves to different humidity conditions during flue-curing. **(A)** Principal component analysis (PCA) analysis of transcriptome profiles from tobacco leaves subjected to two humidity treatments during curing: Treatment 1 (79% RH) and Treatment 2 (93% RH) with temperature maintained at 38 °C. Samples were collected at 6 h, 12 h, and 18 h of curing. **(B)** GO enrichment analysis of differentially expressed genes (DEGs) identified between the two humidity treatments at each curing time point. DEGs were defined using a |log_2_fold change| ≥ 1 and an adjusted *P* < 0.05. **(C)** Chlorophyll a and chlorophyll b contents in tobacco leaves under the two humidity treatments at 6 h, 12 h, and 18 h of curing. Data represent the mean ± SD of three independent biological replicates (n = 3). Statistical significance between humidity treatments at each time point was determined using a two-tailed. Student’s *t*-test. **P < 0.01.

Principal component analysis (PCA) revealed clear separation between samples exposed to moderate humidity (Treatment 1,79% RH) and high humidity (Treatment 2, 93% RH) at corresponding curing time points, indicating distinct global transcriptional profiles in response to humidity differences during curing (**Figure 2A**). Differential expression analysis further showed that the number of differentially expressed genes (DEGs) between the two humidity treatments increased progressively with curing duration, with 373, 703, and 911 DEGs at 6 h, 12 h, and 18 h, respectively (**Figure 2B**).

To gain insight into the functional categories associated with humidity-responsive transcriptional changes, gene ontology (GO) enrichment analysis was performed separately for up-regulated and down-regulated DEGs at each time point (**Figure 2B**). At 6 h, up-regulated DEGs were predominantly enriched in GO terms related to “response to stimulus”, “response to wounding”, and “defense response”. At 12 h, enrichment shifted toward terms including “chloroplast envelope”, “response to salt”, and “response to water”, while at 18 h, up-regulated DEGs were mainly associated with “response to heat”, “chloroplast organization”, and “response to temperature stimulus”.

In contrast, down - regulated DEGs at 6 h were primarily enriched in GO terms related to “protein folding”, “response to unfolded protein”, and “chaperone - mediated protein folding”. At 12 h, these genes were enriched in terms associated with chromatin structure, including “nucleosome”, “nucleosome assembly”, and “chromatin remodeling”. By 18 h, enrichment of down-regulated DEGs was mainly observed in hormone-related categories, such as “jasmonic acid hydrolase”, “regulation of jasmonic acid - mediated signaling pathway”. Overall, down - regulated DEGs were largely associated with protein folding, chromatin structure, and hormone-related processes, whereas up - regulated DEGs were primarily linked to stress responses and chloroplast-related functions during curing. Notably, enrichment of GO terms related to chloroplast structure and organization was first observed at 12 h in response to differences in humidity conditions.

To relate transcriptional changes to physiological outcomes, chlorophyll contents were compared between the two humidity treatments during curing. At both 12 and 18 h, the contents of chlorophyll a and chlorophyll b in tobacco leaves subjected to high humidity (Treatment 2, 93% RH) were significantly lower than those under moderate humidity (Treatment 1, 79% RH) (**Figure 2C**). For instance, chlorophyll a content under high humidity was approximately 62% and 51% of that under moderate humidity at 12 h and 18 h, respectively. These results indicate that high humidity during curing is associated with enhanced chlorophyll loss in tobacco leaves. Based on these transcriptomic and physiological observations, subsequent analysis focused on transcriptional features associated with chlorophyll degradation under humidity conditions.

### Identification of *PAO4* and *CLH6* associated with humidity-related chlorophyll degradation

**Figure 3:**
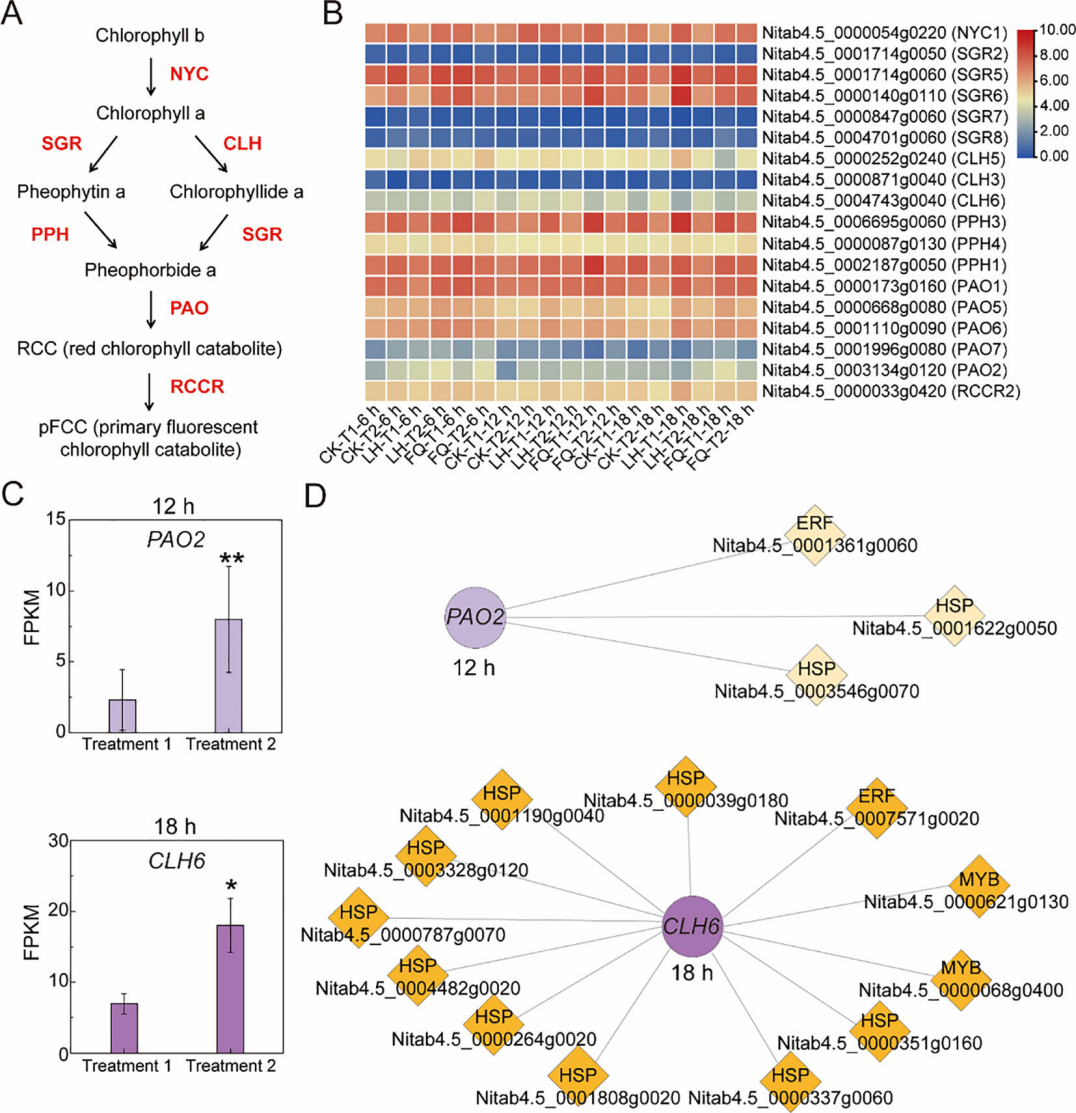
Expression patterns and transcriptional associations of chlorophyll degradation-related genes during tobacco curing. **(A)** Schematic representation of the chlorophyll degradation pathway in plants, showing major enzymatic steps and intermediates. **(B)** Heatmap showing the expression profiles of chlorophyll degradation-related genes across curing samples under different humidity conditions. CK represents the control samples prior to humidity treatment, T1 represents moderate humidity (79% relative humidity, RH), and T2 represents high humidity (93% RH) during flue-curing. **(C)** Relative expression levles of *PAO2* and *CLH6* under T1 and T2 conditions at 12 h and 18 h of curing. Data represent the mean ± SD of three independent biological replicates (n = 3). Statistical significance between humidity treatments at each time point was determined using a two-tailed Student's t-test. * *P* < 0.05; ** *P* < 0.01. **(D)** The regulatory networks of *PAO2* and *CLH6* constructed based on correlation analysis.

Chlorophyll degradation is a stepwise biochemical process in which green chlorophyll molecules are converted into non-colored fluorescent catabolites (pFCC) through the coordinated action of multiple enzymes (**Figure 3A**). To examine transcriptional changes associated with chlorophyll degradation during tobacco flue-curing, we analyzed the expression profiles of 18 structural genes involved in the chlorophyll degradation pathway 54 tobacco samples, including control (CK), Fanqing (FQ), Laohan (LH) leaves under different humidity conditions (**Figure 3B**).

In order to investigate the impact of the single factor of different humidity conditions on the degradation of chlorophyll during the tobacco drying process, we selected the control group for detailed analysis. At the early curing stage (6 h), no significant differences in the expression of chlorophyll degradation-related structural genes were detected between humidity treatments. In contrast, at 12 h, coinciding with accelerated chlorophyll loss, a significant decrease in expression was observed for a PAO family gene (Nitab4.5_0003134g0120, hereafter referred to as *PAO4*). At 18 h, PAO4 expression did not differ significantly between treatment, whereas a CLH family (Nitab4.5_0004743g0040, hereafter referred to as *CHL6*) exhibited pronounced upregulation under high-humidity conditions (**Figure 3C**). These stage specific expression patterns were temporally consistent with the observed dynamics of chlorophyll degradation during curing.

To further characterize transcriptional features associated with *PAO4* and *CLH6* expression under different humidity conditions, correlation-based transcriptional association analyses were performed (**Figure 3D**). For *PAO4*, three transcription factors showed significant correlations with its expression across curing samples (**Supplementary Table S1**). Among them, a heat shock protein (HSP) family gene (Nitab4.5_0003546g0070) displayed the strongest negative correlation with *PAO4* expression (R = -0.91, *P* = 0.01), followed by an ethylene-responsive factor (ERF) gene (R = -0.87, *P* = 0.02).

In the case of CLH6, 12 transcription factors were significantly correlated with its expression (**Supplementary Table S2**). Notably, nine of these belonged to the HSP gene family. The *HSP* gene Nitab4.5_0003546g0070 exhibited a strong negative correlation with *CLH6* expression in response to humidity treatments (**Figure 3D**, **Supplementary Table S2**). Significant correlations were also observed for transcription factors from the MYB, ERF, and NYC families. Among these, the MYB gene Nitab4.5_0000068g0400 showed the highest positive correlation with the *CLH6* expression (R = 0.95, *P* = 0.003) (**Supplementary Table S2**).

Collectively, these results indicate that *PAO4* and *CLH6* display distinct, stage-dependent expression patterns during tobacco curing and are associated with humidity-responsive transcriptional changes. The correlation-based networks constructed here highlight transcription factor families—including ERF, HSF, MYB, NAC, bZIP, MADS, and WRKY (**Figure 3D**)—that are transcriptionly associated with key chlorophyll degradation genes during flue-curing.

### High temperature-associated chlorophyll degradation is linked to *SGR6* and *CLH5* during flue-curing

**Figure 4:**
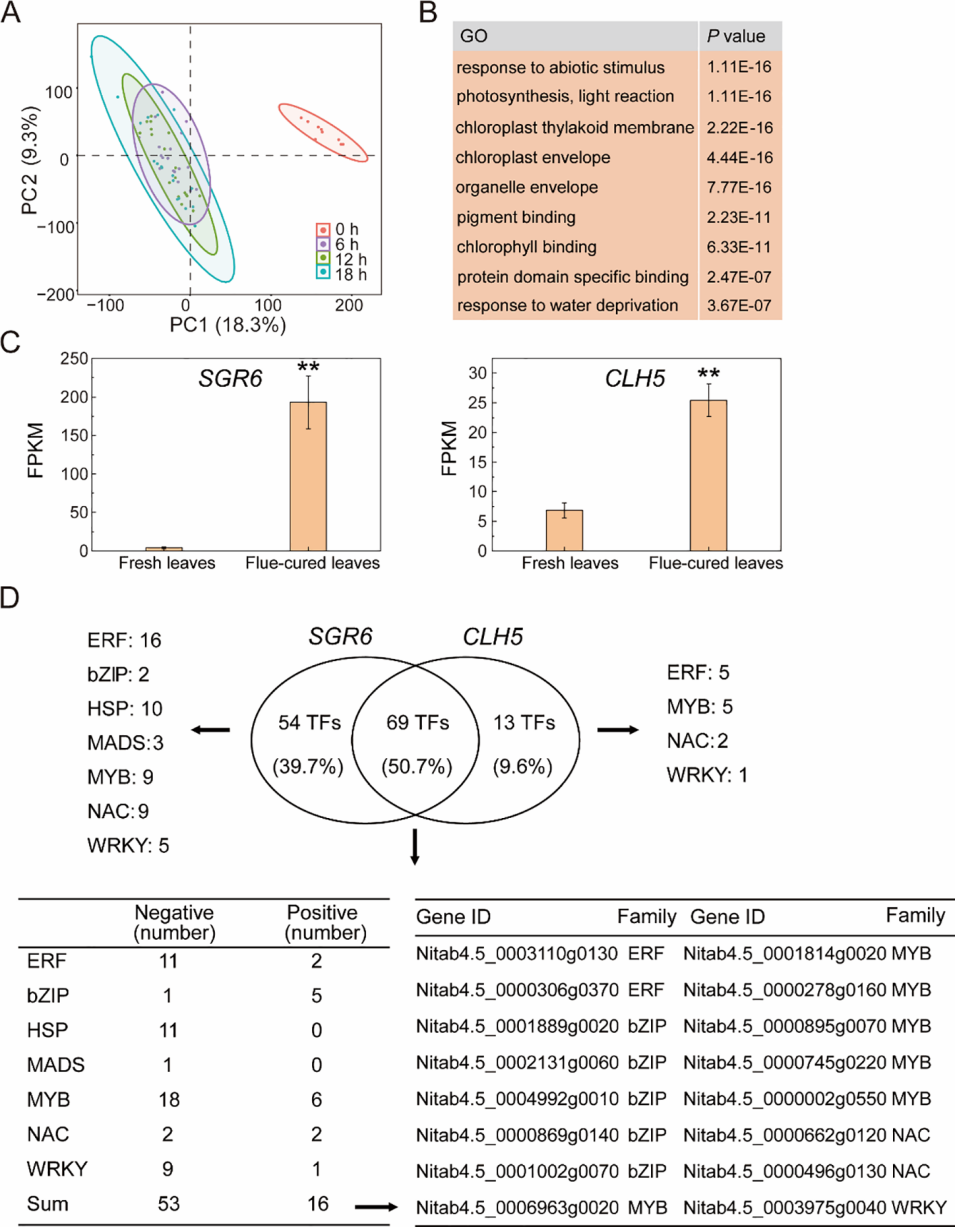
Transcriptomic responses of tobacco leaves to high-temperature conditions during flue-curing. **(A)** PCA analysis of transcriptome profiles from fresh tobacco leaves (0 h) and leaves subjected to flue-curing at 38 °C for 6 h, 12 h and 18 (h) **(B)** GO enrichment analysis of differentially expressed genes identified between fresh leaves and flue-curing leaves at different curing time points. **(C)** Expression levels of *SGR6* and *CLH5* in fresh leaves and flue-curing leaves. and baked tobacco leaves. Data represent the mean ± SD of three independent biological replicates (n = 3). Statistical significance between humidity treatments at each time point was determined using a two-tailed Student’s *t*-test. **P < 0.01. **(D)** Correlation-based transcriptional association networks of *SGR6* and *CLH5* constructed from gene expression profiles during flue-curing.

In addition to humidity, temperature is also a crucial environmental factor influencing chlorophyll degradation during tobacco flue-curing. PCA showed that transcriptome profiles of fresh tobacco leaves collected at 0 h were clearly separated from those of leaves subjected to curing at 38 °C for 6 h, 12 h, 18 h, indicating substantial transcriptional reprogramming during the curing process (**Figure 4A**).

Differentially expression analysis between fresh tobacco leaves and cured leaves identified a large number of DEGs, the number of which increased with curing duration (**Supplementary Table S3**). GO enrichment analysis revealed that these DEGs were significantly enriched in biological processes related to “pigment binding”, “chlorophyll binding”, “chloroplast membrane”, “response to abiotic stress”, as well as “drought - related response” (**Figure 4B**). These results indicate that high-temperature curing is associated with pronounced transcriptional changes in genes related to chloroplast function, pigment metabolism, and stress resistance.

Among the genes involved in the chlorophyll degradation pathway (**Figure 3A**), two structural genes — Stay-Green 6 (SGR) (Nitab4.5_0000140g0110) and Chlorophyllase 5 (CLH5, Nitab4.5_0000252g0240) — exhibited markedly increased expression levels in cured leaves compared with fresh leaves (**Figure 4C**). Notably, the expression of *SGR6* increased by approximately 150-fold during curing relative to the 0 h samples, highlighting a strong association between *SGR6* transcription and high-temperature curing conditions.

To further characterize transcriptional features associated with *SGR6* and *CLH5* expression during high-temperature curing, correlation analysis was conducted between the expression levels of these structural genes and transcription factors. A total of 123 TFs and 82 TFs showed significant correlations with the expression of *SGR6* and *CLH5*, respectively (**Supplementary Tables S3**, **S4**). Subsequently, a VENN analysis revealed that 69 TFs were significantly correlated with both *SGR6* and *CLH5* expression, whereas 54 TFs and 14 TFs were uniquely associated with SGR6 or CLH5, respectively (**Figure 4D**).

Among the TFs associated with *SGR6* and *CLH5* expression, 53 displayed negative correlations and 16 showed positive correlations. These TFs belong to multiple families, including ERF, bZIP, HSF, MADS, MYB, NAC, and WRKY (**Figure 4D**). The gene IDs of TFs positively correlated with *SGR6* and *CLH5* expression are shown in **Figure 4D**, and include representative from the ERF, bZIP, MYB, and NAC families. Together, these correlation-based analyses identify candidate transcription factors whose expression patterns are associated with key chlorophyll degradation genes during high-temperature flue - curing.

## Discussion

The quality of tobacco is largely determined by its chemical composition, which is shaped by both genetic background and postharvest curing conditions. After leaf harvest, environmental factors such as temperature and relative humidity during flue-curing play critical roles in determining the rate and extent of chlorophyll degradation, thereby influencing the visual appearance and overall quality of cured leaves (Chen et al., 2019; Zong et al., 2022; Yang et al., 2023). Although the physiological importance of curing conditions has been well recognized, the transcriptional responses underlying postharvest chlorophyll degradation remain incompletely characterized. In this study, by integrating chlorophyll measurements with transcriptome profiling across 63 tobacco leaf samples, we provide a comprehensive view of how postharvest environmental parameters are associated with dynamic changes in gene expression during flue-curing.

**Figure 5:**
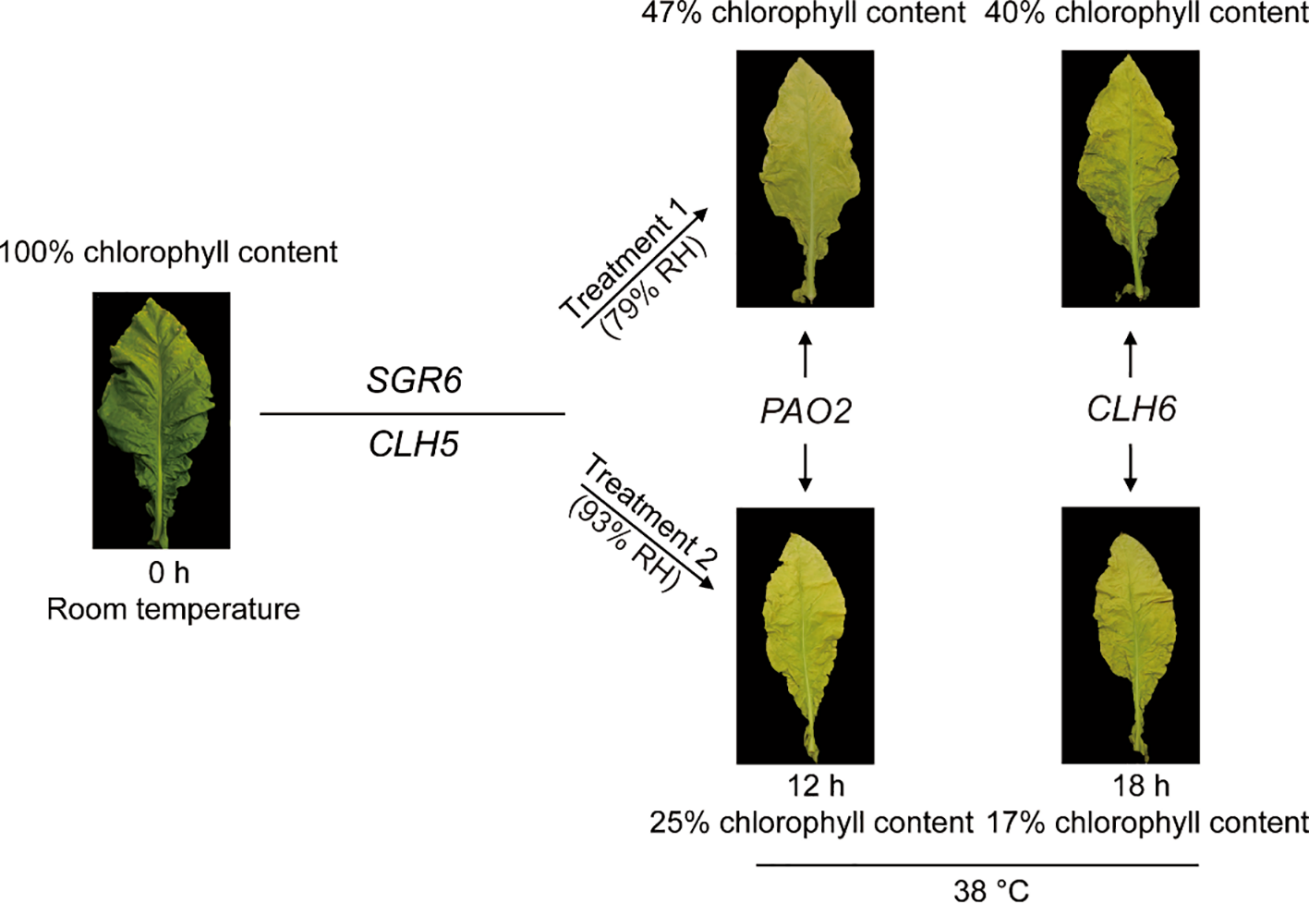
Conceptual model summarizing transcriptional and physiological associations related to chlorophyll degradation in tobacco leaves during flue-curing. This schematic model integrates physiological measurements, transcriptome profiling, and correlation-based analyses to illustrate how changes in relative humidity and temperature during postharvest flue-curing are associated with chlorophyll degradation in tobacco leaves. Elevated humidity and temperature conditions are linked to enhanced chlorophyll loss and distinct transcriptional responses of chlorophyll degradation–related structural genes (e.g., *PAO*, *CLH*, *SGR*) and associated transcription factor families. The model highlights stage-dependent and environment-responsive expression patterns observed during curing but does not imply direct regulatory or causal relationships and is intended as a descriptive framework for future functional studies.

Chlorophyll degradation is a coordinated biochemical process involving multiple enzymatic steps, and the expression of gens participating in this pathway is responsive to both endogenous cues and exogenous factors. Our results demonstrate that chlorophyll degradation during tobacco flue-curing is closely associated with variations in temperature and humidity. Through transcriptome-wide analysis, we identified four structural genes—*SGR6*, *CLH5*, *PAO4*, and *CLH6*—whose expression patterns showed clear associations with curing environments and the temporal dynamics of chlorophyll loss. Notably, *SGR6* and *CLH5* exhibited transcriptional responses primarily associated with high-temperature curing, whereas *PAO4* and *CLH6* displayed stage-specific expression changes linked to humidity conditions (**Figure 5**). These findings suggest that different components of the chlorophyll degradation pathway may be differentially sensitive to postharvest environmental factors.

Previous studies have shown that chlorophyll metabolism can be modulated by preharvest treatments, such as melatonin or brassinosteroid applications, which alter the expression of chlorophyll biosynthesis and degradation genes and subsequently affect leaf maturation and post-curing quality (Wei et al., 2024). In contrast, our study focuses on postharvest environmental regulation and reveals distinct transcriptional response patterns associated with temperature and humidity during curing. Consistent with this distinction, postharvest light exposure has been reported to delay chlorophyll degradation by suppressing CLH activity in lettuce, thereby extending shelf life (Yang et al., 2024). Collectively, these observations suggest that chlorophyll degradation during flue-curing involves transcriptional responses that differ from those occurring during natural senescence or preharvest chemical treatments, highlighting the unique regulatory context of postharvest leaf senescence.

The stage-specific fluctuations in temperature and humidity characteristic of the flue-curing process therefore serve as environmental cues that are the temporally associated with changes in the expression of chlorophyll catabolic genes. These associations point to a phase-dependent coordination of functionally distinct genes during curing, which may channel chlorophyll breakdown through different biochemical routes at successive stages. The observed complexity underscores the sensitivity of chlorophyll degradation to postharvest environmental conditions and reflects the involvement of multiple gene groups rather than a single dominant pathway.

In addition to structural genes, our correlation-based analysis identified several transcription factor (TF) families whose expression profiles were significantly associated with chlorophyll degradation during curing, including HSP, ERF, bZIP, MYB, NAC, and WRKY (**Figures 3**, **4**). These TF families have previously been implicated in stress response, senescence, and temperature or drought tolerance in tobacco and other plant species. For example, overexpression of *HSP70–1* enhances drought tolerance in tobacco (Cho and Hong, 2006), and *HSP70-8b* contributes to thermotolerance (Zhang et al., 2023). Similarly, several NAC family members have been reported to participate in tobacco leaf senescence and stress responses (Li et al., 2018; Wen et al., 2022), and NAC-mediated regulation of jasmonic acid-related pathways has been linked to chlorophyll degradation during senescence (Chang et al., 2024; Lu et al., 2024). While these studies provide useful context, the transcription factors identified in the present work should be considered putative candidates whose involvement in postharvest chlorophyll degradation is inferred from expression correlations rather than direct functional evidence.

Accordingly, further experimental validation—such as dual-luciferase reporter assays, electrophoretic mobility shift assays, or transgenic approaches—will be required to confirm direct regulatory relationships and to establish definitive transcriptional networks governing chlorophyll degradation during curing. In addition, transcriptome profiling revealed that differentially expressed genes were enriched in GO terms related to protein folding, protein maturation, and response to unfolded protein, suggesting that chlorophyll degradation under postharvest stress conditions may occur alongside broader adjustments in cellular proteostasis.

Nitrogen fertilization is another important factor influencing tobacco growth and leaf quality. Although moderate increase in nitrogen supply can alleviate stress and improve yield (Ren et al., 2025), excessive nitrogen application—such as that observed in Laohan and Fanqing tobacco leaves in this study—leads to elevated chlorophyll accumulation in leaves and poses challenges for efficient chlorophyll degradation during curing. Flue-curing consists of three major stages: yellowing, leaf-drying, and vein-drying stages. Our results highlight the yellowing stage as a particularly critical window for postharvest leaf maturation, during which elevated humidity is associated with enhanced chlorophyll degradation. These findings provide practical guidance for optimizing curing conditions to mitigate preharvest quality issues, such as excessive chlorophyll accumulation, while maintaining overall cured leaf quality.

## Conclusion

Our results demonstrate that elevated relative humidity during flue-curing is closely associated with enhanced chlorophyll degradation in leaves, which is beneficial for reducing undesirable regreening frequently observed under environmental stress or high nitrogen fertilization. By integrating physiological measurements with transcriptome-wide analyses, we identified key chlorophyll degradation-related genes and transcriptional features that respond to changes in temperature and humidity during curing process. Importantly, appropriate humidity management during flue-curing was associated with improved degradation of residual chlorophyll without negative affecting cured leaf quality. These findings provide a physiological and transcriptional framework for optimizing curing conditions and offer candidate genes for future studies aimed at developing tobacco varieties with improved curing adaptability.

## Materials and methods

### Plant materials and treatments

Flue-cured tobacco cultivar Hongda (*Nicotiana tabacum* L.), a widely cultivated variety in China with stable agronomic performance and well-characterized curing characteristics, was grown at the Yanhe Experimental Station in Hongta District, Yuxi City, Yunnan Province, China (24.14°N, 102.29°E, 1,635 m altitude). The field experiment was conducted from May to September 2024 under local conventional management practice.

Three nitrogen fertilization regimes were applied in the field. The control treatment received a pure nitrogen application rate of 82.5 kg/ha, corresponding to the standard fertilization level recommended for flue-cured tobacco production. Two high-nitrogen treatments were established to simulate common farmer practices associated with excessive nitrogen input: the Laohan (LH) and Fanqin (FQ) treatments, both of which received a pure nitrogen application rate of 142.5 kg/ha (**Table 1**). Other agronomic practices were kept consistent across treatments.

At physiological maturity, fresh tobacco leaves were harvested from the middle position of plants to minimize variation associated with leaf developmental stage. The harvested leaves were subjected to postharvest curing treatments under controlled environmental conditions. Specifically, leaves were incubated at a constant temperature of 38 °C, corresponding to the initial yellowing stage commonly applied in commercial flue-curing, during which chlorophyll degradation is most active. Two relative humidity (RH) regimes were imposed: Treatment 1 (79% RH) and Treatment 2 (93% RH).

Postharvest treatments were carried out in a programmable environmental test chamber (Memmert CTC256, Kunming, Yunnan, China). Leaf samples were collected at 6 h intervals during curing. Upon sampling, tissues were immediately frozen in liquid nitrogen and stored at -80 °C until further physiological and transcriptomic analyses. The experiment was conducted with three independent biological replicates, each replicate consisting of 15 tobacco leaves collected from different plants within the same treatment.

### Measurement of chlorophyll content

The chemical composition and chlorophyll content were analyzed for samples collected. Chlorophyll content were assessed using the methods outlined in the Chinese tobacco industry standards “Tobacco and tobacco products—Determination of plastid pigments—High performance liquid chromatography method (YC/T382—2010)”. There are three biological replicates for chemical analysis.

### RNA extraction, library construction, and transcriptome analysis

Total RNA was extracted from tobacco leaf samples using the Trizol reagent kit (Invitrogen, Carlsbad, CA, USA) following the manufacturer’s protocol. RNA quality and integrity was assessed by 1.0% agarose gel electrophoresi, RNA purity was evaluated based on the absorbance ratio of A260/A280. Only RNA samples with high integrity and acceptable purity were used for subsequent analyses. After total RNA was extracted, eukaryotic mRNA was enriched by Oligo (dT) beads. RNA library construction and RNA-sequencing were performed by Novogene (Beijing, China).

Raw sequencing reads were subjected to quality control using fastp (v1.21) to remove adaptor sequences, low−quality reads, and reads containing excessive ambiguous bases. Clean reads were then aligned to the *Nicotiana tabacum* genome (https://solgenomics.net/ftp/genomes/Nicotiana_tabacum/edwards_et_al_2017/assembly/) using Hisat2 (2.0.5), with the reference genome index built by the same software. The mapped reads from each sample were assembled in a reference-guided manner using StringTie (1.3.3b). Gene-level read counts were generated FeatureCounts (v1.5.0-p3). Gene expression levels were normalized and expressed as fragments per kilobase of transcript per million mapped reads (FPKM), calculated based on gene length and the number of mapped reads.

### Differentially expressed genes selection, gene ontology enrichment analysis

The clean reads were aligned to the *Nicotiana tabacum* reference genome (solgenomics_nicotiana_tabacum_edwards2017_Ch) with Hisat2 (2.0.5). Gene-level read counts were generated using featureCounts, and only uniquely mapped reads were retained for downstream analyses. Differential expression analysis was performed using the DESeq2 R package, which is based on raw read counts. *P*-values were adjusted for multiple testing using the Benjamini-Hochberg false discovery rate (FDR) method. Genes with an absolute log_2_fold change ≥ 1 and an FDR-adjusted *P* < 0.05 were defined as differentially expressed genes (DEGs).

For expression visualization and comparative analyses, transcript abundance was additionally estimated as FPKM using StringTie (v1.3.3b). Principal component analysis (PCA) was conducted based on normalized gene expression values to evaluate sample clustering and biological replicate consistency. Correlation analysis among biological replicates was performed using the Hmisc R package (https://hbiostat.org/R/Hmisc/). Gene Ontology (GO) enrichment analysis of DEGs was carried out using TBtools-II (Chen et al., 2023), with all expressed genes used as the background set. GO terms with an adjusted P < 0.05 were considered significantly enriched. All transcriptomic analyses were conducted with three independent biological replicates per treatment.

### Statistical analysis

Statistical analyses between two experimental groups were performed using two-tailed Student’s *t*-test in SPSS 19.0 (SPSS Inc., Chicago, IL, USA). Differences with *P* < 0.05 were considered statistically significant. Correlation analysis, differential expression analysis and PCA were conducted using R software.

## Data Availability

The data presented in the study are deposited in the Genome Sequence Archive repository, accession number CRA038071.
