# Supplementary material for: Dynamic transcriptome unveils transcriptional network associated with chlorophyll degradation in tobacco leaves under high humidity and temperature
Source: Front Plant Sci. 2026 Feb 11;17:1758730. doi: 10.3389/fpls.2026.1758730 (PMC12932517; doi:10.3389/fpls.2026.1758730)
Supplement: Supplementary file 2 [file Table2.docx]

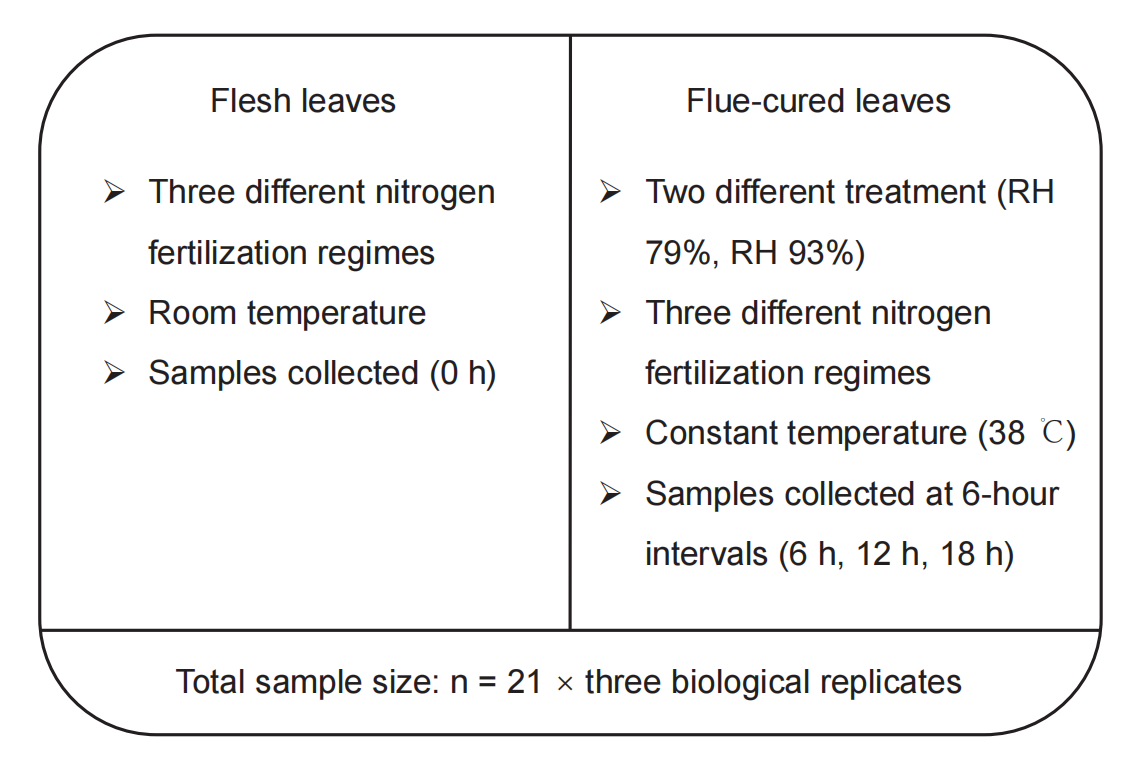


**Figure S1 Schematic overview of experimental design and dataset classification used in this study.**

Tobacco leaves from three tobacco types were subjected to flue-curing under controlled temperature conditions (38 °C) with two relative humidity treatments: Treatment 1 (79% relative humidity, RH) and Treatment 2 (93% RH). Leaf samples were collected at 0 h (fresh leaves prior to curing) and at 6 h, 12 h, and 18 h during the curing process. A total of 63 samples were generated, including nine fresh-leaf samples collected at 0 h and 54 flue-cured samples collected under different humidity conditions. Cured samples were grouped according to humidity treatment, curing duration, and tobacco type, and were used for physiological measurements (chlorophyll content) and transcriptome analyses, including differential gene expression, Gene Ontology enrichment, and correlation-based transcriptional association analyses.
